# Basalt-Fiber-Reinforced Polyvinyl Acetate Resin: A Coating for Ductile Plywood Panels

**DOI:** 10.3390/ma13010049

**Published:** 2019-12-20

**Authors:** Samuel Kramár, Miroslav Trcala, Korawit Chitbanyong, Pavel Král, Buapan Puangsin

**Affiliations:** 1 Department of Wood Science and Technology, Faculty of Forestry and Wood Technology, Mendel University in Brno, Zemědělská 1, 613 00 Brno, Czech Republic; miroslavtrcala@seznam.cz (M.T.); kral@mendelu.cz (P.K.); 2 Department of Forest Products, Faculty of Forestry, Kasetsart University, 50 Ngamwongwan Rd, Lad Yao, Chatuchak, Bangkok 10900, Thailand; korawit.chi@ku.th (K.C.); fforbpp@ku.ac.th (B.P.)

**Keywords:** basalt fiber, FRP, plywood, reinforcement, sandwich panels, layered structures

## Abstract

The aim of this study was to create a reinforced composite wood-based panel that would be leaned towards the environment Plywood was used as a core material and fiber-reinforced polymer was used as a reinforcement. Conventional resin for the fiber-reinforced polymer was substituted with polyvinyl acetate (PVAC), which has several advantages, such as a lower price, easier handling, and better degradability. The second chosen component, basalt fiber, is cost attractive and environmentally friendly. The combination of one and two layers of fabric with three fiber fractions and 4 mm thick plywood was investigated. The best results were achieved with two layers of fabric and the highest fiber fraction. The improvements of the ultimate bending load and bending stiffness of the plywood in the perpendicular direction were 305% and 325%, respectively. The ultimate load and stiffness of the parallel direction were improved by 31% and 35%, respectively. However, specimens always failed in the compressional zone. The highest reinforcing effect was found with the impact test: The energy required to fracture specimens increased by 4213% and 6150% for one and two layers of fabric, respectively. In conclusion, specimens exhibited high ductility due to the PVAC and basalt fiber. The amount of work and energy required to cause fractures was extensive.

## 1. Introduction

Plywood is a material that was used mostly in the past for aircraft, cars, and sports goods [1]. It has a successor. The fiber-reinforced polymer (FRP) is a composite material now used for these high-tech products [2], due to FRP’s superior properties when considering both mechanical and physical aspects. However, environmental problems connected with FRP are gaining attention [3]. In this respect, plywood which is made of sustainable material [4] is better than FRP. The drawbacks of these materials could be lessened and plywood could be well reinforced if they are properly combined and modified into one composite material.

Currently, most FRPs use epoxy or polyester resins as a matrix [5]. However, the reinforcement of plywood could allow the mounting of fabrics with other wood-working adhesives. Some of these adhesives are less harsh for the environment than conventionally used resins. One of these is the polyvinyl acetate (PVAC) adhesive. For example, it is used instead of harmful urea-formaldehyde (UF) resin for particleboard production [6], and it could also substitute for the polymeric matrix of FRP. It is worth mentioning that wood processing for wood-based panels has less influence on life cycle impact, while formaldehyde-based chemicals have the highest one. On the other hand, the impact of PVAC is less significant when using these panels for products such as doors [7]. The advantages of PVAC adhesive over epoxy resin are its lower price, faster solidification, easy handling and cleaning, and its less negative impact on the environment [8]. Moreover, PVAC is degradable, and some types are even biodegradable [9,10]. Still, epoxy resin has a higher modulus of elasticity and better water resistance [11]; thus, application of PVAC will not necessarily increase stiffness. Additionally, PVAC is not soluble in water but swells due to its presence. As a consequence, this leads to reduced durability [9]; these panels should not be directly exposed to rainfall.

Another component of FRP is fibers. Commonly used fibers are made of carbon, aramid, or glass [12]. These are connected with either high production costs [13] or problematic recycling [14]. For example, carbon fiber manufacturing requires much energy [15]. Production of glass fibers requires additives. This is a tangible difference from basalt fibers. Basalt is directly processed into fibers by melting without the need for any modification [16]. The melting temperature of basalt fibers is only slightly higher than that for glass fibers, but its mechanical properties are also higher [17]. Life cycle assessment (LCA) of overall basalt fiber production confirms a considerably lower environmental footprint than those of carbon or glass fibers [18]. Moreover, Inman et al. [19] found that 86.6% of emissions in basalt-fiber-reinforced polymer (BFRP) production is caused by epoxy resin. Recycling of basalt fibers is notably eased because basalt as the material is not modified. However, recyclability is a function of the application, not just the material. Still, a water semi-resistant, thermo-plastic polymer provides more options for the removal of reinforcing layers and separation of basalt fibers. Additionally, sources of basalt are abundant [20]. Nowadays, basalt fibers are of interest for reinforcement of wood–plastic composites [21] and reinforcement of timber beams [22,23].

Plywood and other wood-based panels were successfully reinforced with some types of FRP. One of these cases is a carbon fiber and phenol-resorcinol formaldehyde matrix. However, specimens tested by bending failed due to delamination [24]. Authors explain this behavior with the distinctive mechanical properties of the materials used. Delamination also occurred when strengthening plywood with basalt-fiber-reinforced epoxy resin [25]. Several other studies describe their approach to the reinforcement. Carbon fibers bonded by isocyanate-based adhesive were used by Ashori et al. [26]. A similar study with chopped carbon fibers and phenol-resorcinol formaldehyde was performed by Heng et al. [27]. Bal et al. [28] reinforced plywood with glass fibers and phenol-formaldehyde adhesive. Another type of study is of strengthening with glass-fiber-reinforced polyester resin [29]. These were applied both between veneers and on the surfaces of the plywood. Most of these studies describe a significant improvement in terms of stiffness, flexural strength, and ultimate failure load. The best effect was usually achieved by a higher amount of fibers, with their position closer to the surface of the panel. However, these studies have another feature in common. The matrices used have either high amounts of hazardous volatile organic compounds or contain toxic substances; basalt fibers are scarce in this up-to-date research on wood-based panel (WBP) reinforcement.

PVAC resin alone cannot be used for FRPs due to its low stiffness [30] and volume-loss-related solidification. However, it stands a fair chance when mounted on the plywood with basalt fibers. All of these materials could be combined into strong panels that resist tension failure and the impact load. Moreover, these panels should be more environmentally acceptable with advantageous costs.

## 2. Materials and Methods

### 2.1. Panel Production

A reinforcing layer was applied to the plywood surface. The main goal was to improve its mechanical properties when subjected to quasi-static three-point bending as well as impact load. For this purpose, reinforcement of any timber product or WBP (wood-based panel) should be mounted at least on its tensional side, where fracture usually occurs [31]. However, for plywood, the symmetrical composition should be maintained [32]. For this reason, both surfaces of plywood were covered with basalt fabric embedded in the PVAC matrix.

**Figure 1:**
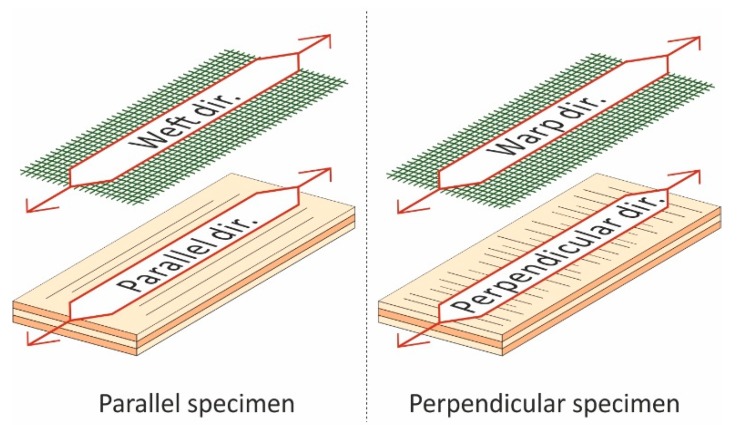
Orientation of fabric in relation to the plywood direction. Sample of flexural specimens.

For the experiment, 4 mm thick, three-layered birch plywood (AZ wood a.s., Brno, Czech Republic) was used. Twilled basalt fabric (Basaltex a.s., Šumperk, Czech Republic) was bonded with Ponal Super 3, D3 PVAC adhesive (Henkel s.r.o., Prague, Czech Republic). Two compositions with either one or two layers of fabric were produced. Raw plywood was used as a control panel. Weft and warp directions of the fabric were collinear with the parallel and perpendicular directions of the plywood, respectively (Figure 1).

Both groups of plywood with single and double coating were fabricated with three different adhesive amounts. Each adhesive amount was calculated to give a specific fiber fraction in the solidified PVAC adhesive. At first, the thickness of the fabric was measured according to ISO 4603 (1993) [33]. The average thickness of the basalt fabric was 0.22 mm; thus, the bulk volume (with voids) of one square meter of fabric was 220 cm^3^. The areal weight of the fabric was 340 g·m^−2^. This was taken from the product datasheet. The mass (m) of 340 g—which is the weight of one square meter of fabric—and the bulk volume (V) of this one square meter of fabric were used to calculate its bulk density (ρ) according to Equation (1). The thickness of the fabric was considered to be a height when calculating the bulk volume.

(1) $$\rho =\frac{m}{V}$$

The bulk density (contains both basalt fibers and voids) of the fabric was 1.55 g·cm^−3^. It was compared with the density of solid basalt rock at 2.8 g·cm^−3^ [34]. Therefore, the volume of fibers in one square meter of fabric was approximately 121 cm^3^. This amount represents 55% of the bulk fabric volume and is considered to be an upper limit for the fiber volume fraction in this fiber-reinforced PVAC resin. Additionally, variants with lower fiber fractions of 50% and 45% were to be fabricated, as this could affect the final mechanical properties [35]. This fiber fraction represents a theoretical ratio of the volumes between fibers and solidified PVAC.

The exact mass of wet adhesive to achieve these fiber fractions had to be calculated. The fiber volume of 121 cm^3^ was considered to be 55%, 50%, and 45% fiber volume fraction in the reinforcing coating. Thus, the volume of the PVAC adhesive had to be 99, 121, and 143 cm^3^ for these fiber fractions after solidification, respectively. At first, the required mass of the solidified adhesive was estimated. The density of the solid PVAC was 1.2 g·cm^−3^. The exact mass of the Ponal super 3 PVAC that was supposed to be achieved in the solidified state was estimated by multiplication of its volume with its density, both of the solid phase (Equation (1)). This was done for all three fiber fractions.

However, this value has to be the weight of the solid-state adhesive that is applied in a wet state. Therefore, mass lost must be compensated, since PVAC solidifies due to evaporation—release of water. This can be simply done by dividing the mass of solidified adhesive by its solid content. Solid content is a mass of solid particles that form solidified adhesive when the water has evaporated. In the case of Ponal super 3, solid content particles share 50% of the wet adhesive mass.

Finally, the amount of applied wet adhesive was 238, 291, 356 g·m^−2^ that, according to this theoretical evaluation, represent 55%, 50%, and 45% fiber volume fraction, respectively. This amount was used for each layer of basalt fabric. The control plywood is abbreviated as P. Variants marked as 1 or 2 refer to the amount of fabrics on each surface of reinforced plywood, and the additional numbers 55, 50, and 45 refer to the fiber volumetric fraction. The design of the experiment is summarized in Table 1.

The stacking sequence for one layer of reinforcement was an application of 2/3 of the total adhesive amount per layer on the plywood surface with a hand roller. The basalt fabric was applied. Finally, the rest of the adhesive was spread on the surface of the composite. The laminating sequence for a double layer of basalt fabric was an application of 1/3 of the adhesive amount per double layer on the surface of the plywood. Then, the basalt fabric was mounted. An additional 1/3 of the adhesive with the second layer of basalt was applied the same way as the first one. Finally, the rest of the adhesive was spread on the panel surface. Adhesive amounts were used as calculated for each fiber fraction.

According to EN 315: Standard for tolerances and dimensions of plywood, 4 mm thick panels may vary from 3.5 to 4.3 mm [36]. Hence, smaller panels for control plywood, plywood with reinforcement with one layer, and plywood with reinforcement with two layers of fabric were cut from one panel only. Otherwise, the thickness variation could have a negative influence on the results among variants of each group.

**Figure 2:**
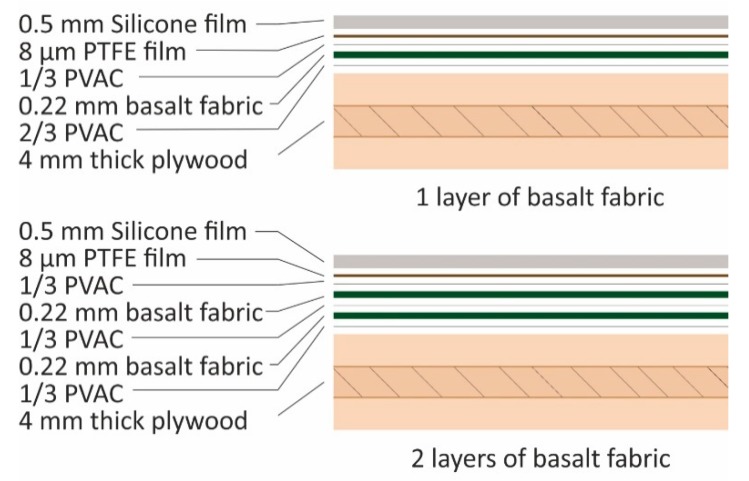
Stacking sequence of panel production. Stacking of only one surface is illustrated, but reinforcing layers were placed symmetrically on both surfaces.

The composites were covered with 8 μm thick high-gloss PTFE (polytetrafluoroethylene) separator (Tart s.r.o., Brno, Czech Republic). A 0.5 mm thick silicon layer (Gumex s.r.o., Brno, Czech Republic) was placed on the very top. The first PTFE created a glossy surface on the pressed composite. The silicone layer adapted to the roughness of basalt fabric and provided fewer gaps and more even pressure throughout the structured fabric. The stacking sequence is presented in Figure 2; however, only one surface is shown for simplicity.

**Figure 3:**
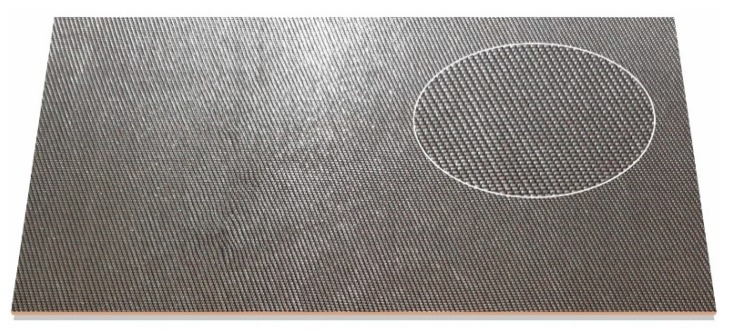
Plywood with basalt fabric and glossy surface. A magnified section is highlighted.

The composites with one and two layers of basalt fabric were pressed for 20 and 30 min, respectively. The pressing temperature of 100 °C and a pressure of 1.5 MPa was the same for both compositions. The resulting panels had fine glossy surfaces with fabric structures (Figure 3).

### 2.2. Testing of Panel Properties

The composite panels were investigated for ultimate load and stiffness (EI) in bending according to the EN 310 (1990) standard [37] by ZH050/TH 3A universal testing machine (Zwick Roell AG, Ulm, Germany). *EI* was calculated in Nmm^2^ according to Equation (2), where *F_2_ − F_1_* is an increment of applied load on the linear part of the load-deflection curve, *w_2_ − w_1_* is an increment of deflection corresponding to *F_2_ − F_1_*, and *l* is a span in bending.

(2) $$EI=\frac{l^{3}\left(F_{2}-F_{1}\right)}{48\left(w_{2}-w_{1}\right)}$$

The impact test was performed on a DPFest 400 impact machine (Labortech, Opava, Czech Republic) following the ISO 6603 [38] standard. Two panels per variant were made. Ten parallel and ten perpendicular specimens (Figure 1) were cut from each panel for the flexural test, amounting to 20 specimens per variant and orientation. Sixteen specimens per variant were cut for the impact test.

All data were subjected to statistical testing. The data normality was evaluated by the Shapiro–Wilk test. Levene and Brown–Forsythe tests were used to prove the homogeneity of variances. ANOVA (analysis of variances) was used under fulfilment of homoscedasticity. Significant differences between groups were compared by the Tukey HSD (honestly significant difference) test. A nonparametric Kruskal–Wallis ANOVA with multiple comparisons of p values was chosen for the data groups that did not have normal distribution or homogeneous variances. All data were tested at a 95% confidence level that represents 0.5 probability value (*p*). Results of the ANOVA analysis are marked in each relevant figure and table. However, only the significant difference compared to the control (reference) plywood is provided.

A material model was created for a deeper understanding of results. Its main contribution was to show the stress distribution through the panel in the linear portion of load and deflection. At first, the fabric was tested according to ISO 4606 (1995) [39] in order to determine its tensional properties in the warp and weft directions. The test was carried out on a ZH050/TH 3A universal testing machine. The tensile moduli of elasticity were 40 and 37.9 GPa for the warp and weft directions, respectively. Material constants for birch plywood were obtained from the Handbook of Finnish plywood [36]. The data for PVAC were used by Konnerth et al. [11]. The final results were used to describe the control plywood and reinforcement with one layer of fabric. The model was based on a numerical solution by the finite element method (FEM). The plywood and the reinforced plywood were calculated as layered (laminated) shell structures. Deflection and stress analyses were performed; the calculation considered shear coupling according to the laminate Mindlin–Reissner theory. In this theory, the normal to the mid-surface remains straight, but is not necessarily perpendicular to the mid-surface [40].

## 3. Results and Discussion

### 3.1. Density

**Figure 4:**
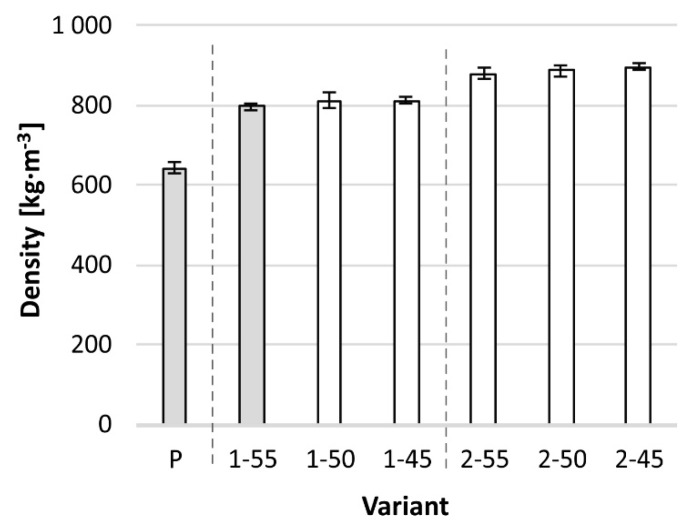
Density of all variants. White columns are significantly (*p* ≤ 0.05) different from control plywood P.

The reinforced panels exhibited increased density (Figure 4), which can generally be attributed to the PVAC adhesive and basalt fibers [34]. Both had a considerably higher density than that of birch plywood [36]. Statistically, the 1–55 variant is not significantly different (*p*-value = 0.7) compared to the control plywood. The 1–50 and 1–45 are significantly different, with *p*-values of 0.008 and 0.005, respectively. Two-layered reinforcement caused a highly significant increase in density, leading to the *p*-value of 0.000 for all three variants compared to the control plywood.

Variants with one layer of fabric had a notably higher increase in density compared to the reference panels, while the difference with two-layered variants was lesser. There are three possible reasons for these results. First, when more layers are added, the be density would be marginally closer to that of the PVAC–basalt fiber composite. Hence, panel density would be less and less different with each added layer. The second reason is the variable thickness among plywood panels, which causes the non-proportional change of density. Finally, hot pressing with added moisture from the adhesive could slightly plasticize the panel, which can lead to densification [41]. The amount of adhesive affected panel compression. Groups with one and two layers of reinforcement manifested a thickness decrease as the amount of adhesive rose. However, the average decrease of all variants was 42 μm from 55% to 50% fiber fraction, and only 4 μm from 50% to 45% fiber fraction, which are quite insignificant dimensional changes.

### 3.2. Ultimate Bending Load and Bending Stiffness (EI)

Parallel specimens have grains of surface veneers aligned with their longer sides (Figure 1). These give them higher moduli of rupture (MOR) because the longitudinal tension and compression strength of wood are always superior to its strength in the perpendicular direction. The same applies to the modulus of elasticity (MOE) [42]. The grain orientation of perpendicular specimens is shifted by 90°. These specimens represent two main axes of plywood. Mechanical properties in these axes may vary greatly [43]. The fewer plies are used, the more different the mechanical properties will be. The highest difference is in the least homogeneous three-plied plywood [36].

The ultimate load and stiffness of all variants in the parallel direction are presented in Table 2. The highest values were observed in the plywood with two layers of reinforcement. Overall, the addition of reinforcing layers had a beneficial effect. Moreover, none of the reinforced specimens failed in the tension side, which is the usual location of the fracture.

The statistical comparison of variants determined that one layer of reinforcement does not significantly increase the ultimate failure load. The *p*-value was higher than 0.8 for all variants with one layer of basalt fabric. All variants with two layers of reinforcement were significantly different compared to control plywood at the *p*-value of 0.13. Similar results were obtained for bending stiffness. One layer of reinforcement did not significantly (*p* > 0.1) increase EI, while two layers had a highly significant effect (*p* = 0.000) on EI increase.

**Figure 5:**
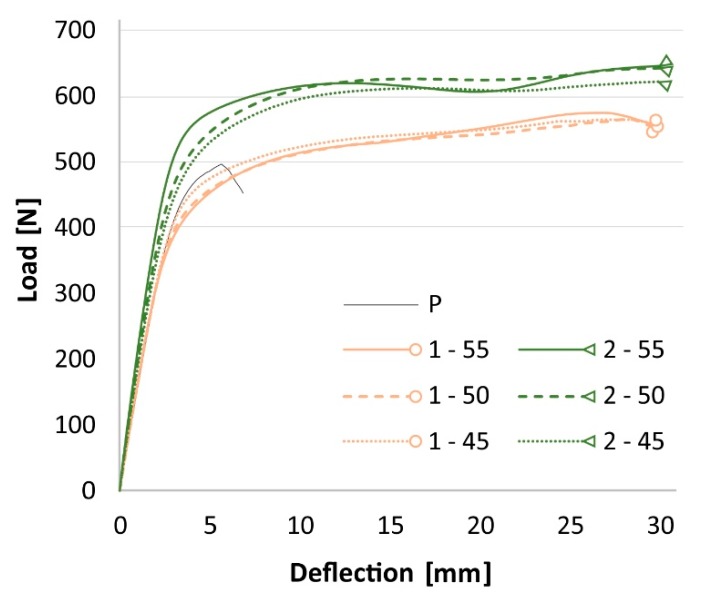
Behavior of parallel specimens during the three-point bending test.

Load-deflection behavior (Figure 5) was different compared to the control plywood. Specimens yielded and deflected with a slightly rising force. Finally, the test was aborted at 30 mm deflection, which was the limit of the extensometer. The highest increases of the ultimate load and EI compared to control plywood were 31% and 35% for the 2–55 variant, respectively.

**Figure 6:**
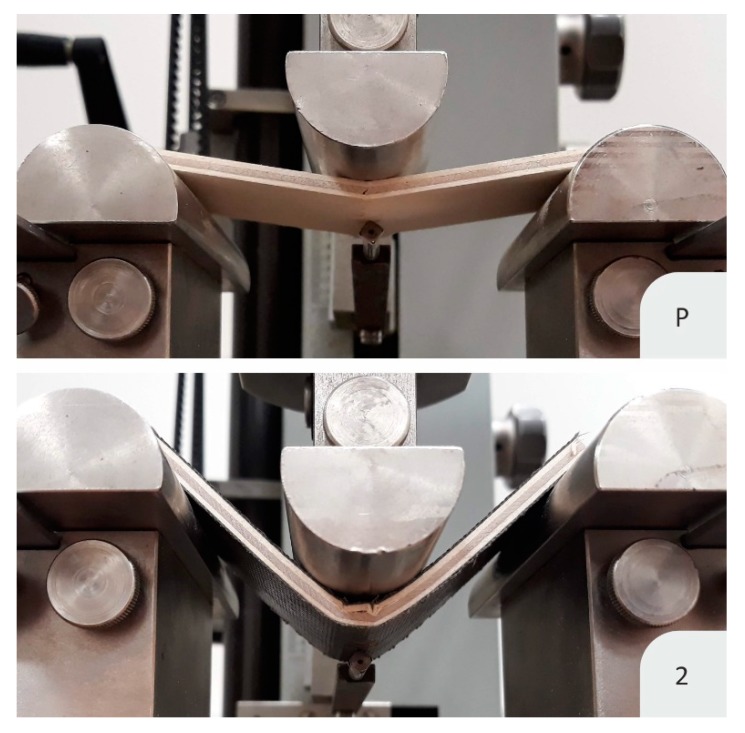
Deflection and failure of a control specimen (P) and a specimen with two layers (2) of basalt-fiber-reinforced PVAC. The reinforced variant exhibited compression failure.

The higher ultimate load and EI are caused by the increased thickness and density [44] between main groups, but also due to the high tensile strength and modulus of basalt fibers. Compression yield was the first to occur because the compressive yield strength of wood is only half of the tension. Low compression yield strength leads to a lower modulus of rupture (MOR) and modulus of elasticity (MOE) of flexure-loaded timber elements [45]. The PVAC matrix is problematic, as it is less rigid than the conventional resin for FRP [30]. Overall, the contribution of basalt-reinforced PVAC on the compression side is low, since the MOEs of PVAC vary between 0.5 and 4 GPa [30], while the MOEs of 4 mm thick birch plywood are approximately 10.7 and 6.8 GPa for the parallel and perpendicular directions, respectively. Thus, the increases in thickness and density were not fully reflected in increased load-bearing capacity and EI. This can be seen with the two-layered reinforcement (Figure 6) in the parallel direction. The compression failure indicates that the weakness of this composite is located on its top. The matrix is the part that withstands the compression load of FRP composites; epoxy resin has an MOE approximately ten times higher than that of wood [46]. Therefore, fiber-reinforced epoxy would be better for the compression side of plywood, but it would not correlate with the environmental aspect of this study.

**Figure 7:**
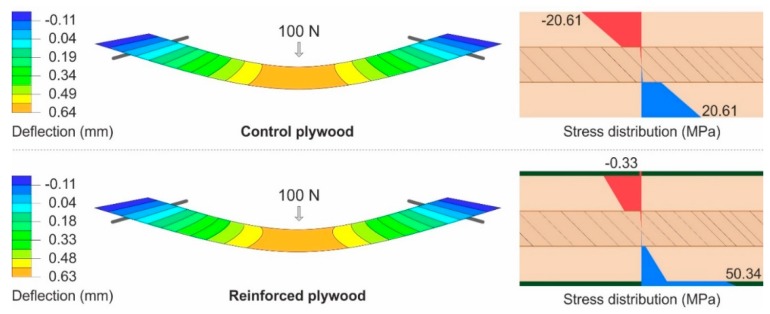
Deflection and stress distribution of parallel specimens at a 100 N load.

On the other hand, the matrix has a negligible effect on the tensile strength of FRP [35]; basalt-fiber-reinforced PVAC is a suitable reinforcement for the tension side. Additionally, PVAC is the least brittle among wood bonding adhesives; it has the ability to sustain high deformations without fractures [11] and a high creep factor. Therefore, it adjusts to dimensional changes in the wood [47]. Figure 7 shows the stress distribution of parallel specimens. The model also confirms low stress on the compression side of plywood reinforced with one layer of fabric, while the tension side was properly involved and bears a high portion of the stress.

In general, plywood has much lower MOR and MOE in the perpendicular direction. This is the direction that should be strengthened to create a more homogeneous panel. The tensile strength of wood in the perpendicular direction can even be 40 times lower than its strength along the grain [48]. Thus, reinforcement with basalt fibers had a more positive effect. Again, the best effect was found with two layers of fabric and the highest fiber fraction. The ultimate load and EI in the perpendicular direction increased by 305% and 325%, respectively (Table 3). The directions of the plywood were made more uniform. Therefore, this type of reinforcement can be used for more demanding applications in the transportation industry—for example, such as for linings of containers or vans [29].

The ultimate failure load was significantly increased (*p* < 0.006) with one layer of reinforcement. When two layers were used, only 2–55 had a significant effect (*p* = 0.027). The other variant did not have this effect, mainly due to higher standard deviation. On the other hand, the two layers of reinforcement had a significant influence on the EI increase (*p* < 0.005) with all variants, while only the 1–55 variant caused a significant (*p* = 0.000) increase of IE among one-layered reinforcements.

**Figure 8:**
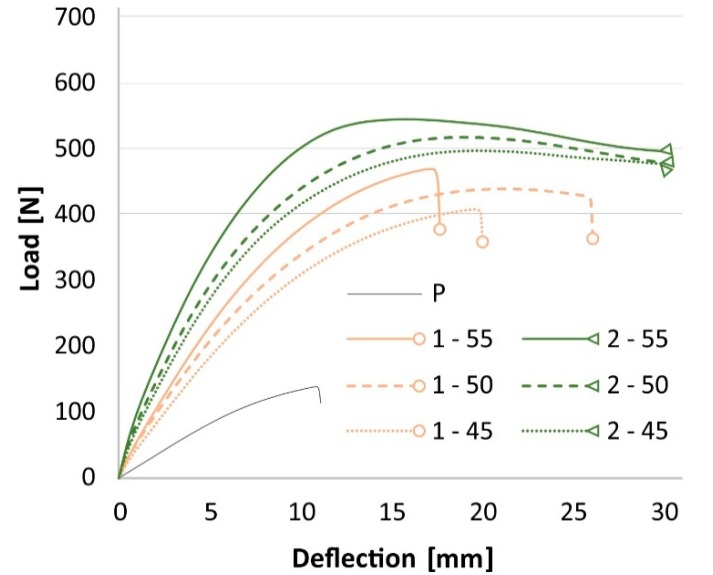
Behavior of perpendicular specimens during the three-point bending test.

Perpendicular specimens had a distinctive behavior during the bending test. All specimens with one layer of fabric exhibited a tension failure of the bottom reinforcement, which is shown as an abrupt drop in the load-deflection curve (Figure 8).

**Figure 9:**
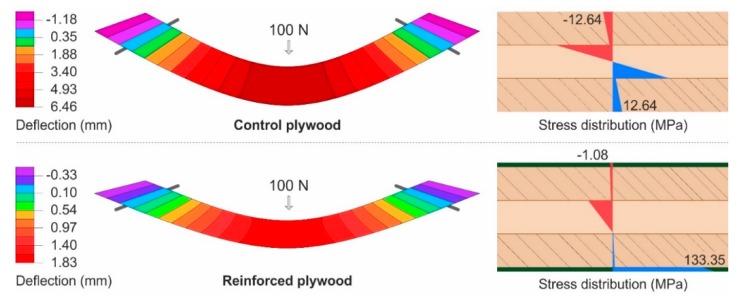
Deflection and stress distribution of perpendicular specimens at a 100 N load.

The reinforcement of parallel specimens was supported by the strong veneer, which bore a part of the load. The reinforcement of perpendicular specimens took most of the load, because the surface veneer in this direction had very low strength (Figure 9). Therefore, the specimens failed prior to 30 mm deflection and the abortion of the test. These results suggest that two layers of reinforcement are convenient. However, considering the results in both directions and the production costs, the reinforcement with one layer of basalt fabric embedded in PVAC resin has its advantages too.

Three volume fiber fractions were tested for the one and two symmetrical layers of basalt fiber–PVAC reinforcement. Some voids may have occurred during solidification of the adhesive due to its shrinking. However, small voids have a minimum effect on the mechanical properties of FRP [13]. The estimated fiber volume fractions were 55%, 50%, and 45%. FRP is known to have better mechanical properties with higher fiber volume fractions. The same trend was observed with almost all variants. With either one or two layers of reinforcement, mechanical properties were increased. However, the overall thickness of the panel decreased with a higher amount of adhesive. This is in contradiction with common FRP, because the lower fiber fraction means the same number of fibers, but a larger cross-section of the composite [49]. In the density section, the thickness decrease in one group was related to higher amounts of PVAC. Probably, only the thickness of the plywood was reduced, because basalt fibers cannot be affected by moisture or a temperature of 100 °C [16]. As a consequence, thinner specimens could be bent more easily. Overall, reduction in panel thickness led to lower load-bearing capacity and stiffness.

### 3.3. Impact Strength

The control plywood subjected to impact load by a falling object had visible fractures at low impact energy. On the other hand, reinforcement with one layer of fabric required 40 times more energy to cause a visible fracture. With two layers of fabric, it was almost 62 times more (Table 4). The evaluated data can be statistically categorized into three homogeneous groups. Those are control plywood, panels reinforced with one layer of fabric, and panels reinforced with two layers of fabric. The data of panels with one layer of reinforcement had *p*-values of less than 0.03 compared to the control plywood. Panels with two layers of reinforcement even return *p*-values of 0.000, indicating a highly significant difference compared to the control plywood. All in all, reinforcement always caused significantly increased resistance to impact energy.

**Figure 10:**
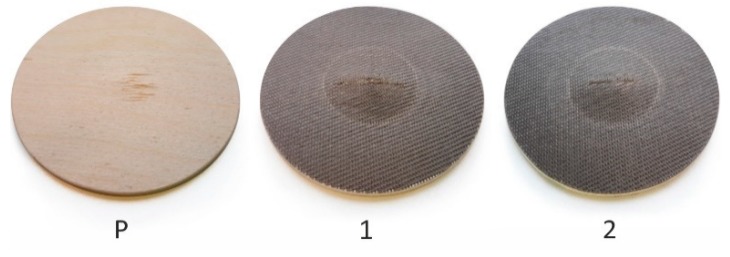
Fractures of specimens subjected to testing by impact load. Control plywood (**P**) and reinforcement with one (**1**) and two (**2**) layers of fabric.

A disadvantage of plywood is its layered construction. Each layer has one direction with low strength. Penetration through such plywood, especially a thin one, is possible, as veneers may fail one by one in an independent manner that is limited mainly by the shear strength of the bond-line. The main contribution of the basalt fabric was its structure. It is made of warp and weft threads woven together. The reinforcing coating was more homogeneous, threads interacted with each other [50], and this layer behaved uniformly in both the warp and weft directions, thus reinforcing both the parallel and perpendicular directions of plywood. However, the real reason for such a significant improvement in resistance to impact strength is the fixed positions of the specimens. This is a difference from three-point bending, because specimens could not be deflected as much as those that were freely placed. Then, the tension strength of basalt fibers could be utilized. The basalt fibers—which have a tensile strength approximately 10 times higher [51,52,53] than that of birch wood [48] in its longitudinal direction—took effect. Thus, tension failures were restricted and the PVAC provided high ductility (Figure 10). In the reinforced specimens, a narrow failure where the fabric could not withstand the tension load can be seen.

Comparing the types of specimens for the impact test and three-point bending test shows an interesting result. Only warp or weft threads bore the load during three-point bending, and specimens could be deflected as a result of the low stiffness of the PVAC. The impact test shows the benefit of ductile adhesive and basalt fibers. Plywood loaded by impact force is supposedly not tested only in the one direction; stress and strain are dispersed over the whole panel, where the weakest spot is prone to fail. However, the ductile adhesive does not allow delamination due to these forces; compared to veneer, basalt fabric provides great resistance against failure in all directions. It seems that due to the high deflection, the stress may shift mainly to tension, where basalt fibers can grant their potential. This behavior suggests that these panels should be constrained in the place of their final application.

Reinforced plywood is not suitable for structural applications. This is mainly due to the thermoplastic properties of PVAC resin [54]. However, there are several suitable applications. Panels with low weight and high resistance to impact load could be used for packaging, substituting for boxes made of thicker wooden panels. The described technique of fabric application is also suitable for other wood-based panels due to the versatility of PVAC resin. Therefore, this kind of reinforcement might find use in additional products such as safety doors.

## 4. Conclusions

A new method of plywood reinforcement was investigated. It seems to be a feasible alternative to the current state of research. Many current methods use stiff resins for structural applications. Their disadvantage is either cost, negative environmental impact, or sometimes even detrimental compatibility with wood or reinforcing fibers. For the purpose of reinforcement, the PVAC seems to be an attractive alternative, as it is a rather ductile polymer that adapts to dimensional changes and does not delaminate due to higher shear stresses.

Reinforced plywood panels that were tested by three-point bending improved both in ultimate failure load and stiffness. This effect was to a lesser extent in the parallel direction, while mechanical properties increased more than threefold in the perpendicular direction. However, in most cases, failure was not reached at the level of the maximum tensile stress. Rather, the compression yield allowed a high deflection of the specimens with increased resistance to load, which required a high amount of work compared to the control plywood.

The problem of deflection was solved during the impact test, and it was found that 40 and 62-times more energy were required to cause visible fractures. The testing tool fixed circular specimens all around. Thus, the deflection was restricted. The reason for this improvement is the combination of basalt fibers and PVAC adhesive. The basalt fabric constrained tensile failures and the PVAC contributed to the high ductility of the whole composite panel while being resistant to shear failure.

These panels, however, pose some limitations due to their degradability by both biotic and abiotic factors. Therefore, their area of application must exclude direct exterior exposure. Some limitations are also dependent on the thermoplastic properties of these panels. This product, however, would have a good resistance against the impact force during standard service conditions.

## Figures and Tables

**Table 1:** Design of the experiment.

Variant	Number of Reinforcing Layers *	Fiber Fraction [%]	n_1_	n_2_
P	0	n/a	20	16
1–55	1	55	20	16
1–50	1	50	20	16
1–45	1	45	20	16
2–55	2	55	20	16
2–50	2	50	20	16
2–45	2	45	20	16

* Reinforcing layers were placed symmetrically on both surfaces. n_1_ = number of specimens for the three-point bending test. n_2_ = number of specimens for the impact test.

**Table 2:** Flexural properties in the parallel direction.

	Ultimate Load [N]			EI [×10^3^ Nmm^2^]		
Variant	Mean	St. Dev.	ANOVA	Mean	St. Dev.	ANOVA
P	502	25.6		3355	212	
1–55	537	29.5		3698	236	
1–50	563	31.0		3443	157	
1–45	570	29.5		3443	121	
2–55	658	42.0	***	4527	130	***
2–50	651	45.8	***	4112	355	***
2–45	636	29.7	***	3937	229	***

*** significant difference (*p* ≤ 0.05) compared to control group P.

**Table 3:** Flexural properties in the perpendicular direction.

	Ultimate Load [N]			EI [×10^3^ Nmm^2^]		
Variant	Mean	St. Dev.	ANOVA	Mean	St. Dev.	ANOVA
P	135	21.5		339	45	
1–55	465	24.0	***	896	56	***
1–50	435	14.8	***	795	26	
1–45	415	13.7	***	754	33	
2–55	545	24.0	***	1441	60	***
2–50	513	31.4		1215	110	***
2–45	510	30.4		1234	141	***

*** significant difference (*p* ≤ 0.05) compared to control group P.

**Table 4:** Impact energy that caused visible fracture of specimens.

	Impact Energy [J]		
Variant	Mean	St. Dev.	ANOVA
P	0.5	0.1	
1–55	20.0	1.4	***
1–50	19.9	0.9	***
1–45	19.9	1.5	***
2–55	30.9	1.2	***
2–50	30.7	1.3	***
2–45	30.8	1.0	***

*** significant difference (*p* ≤ 0.05) compared to control group P.
